# Diagnostic Performance of Serum IgG4 Levels in Patients With IgG4-Related Disease

**DOI:** 10.1097/MD.0000000000001707

**Published:** 2015-10-16

**Authors:** Kuang-Hui Yu, Tien-Ming Chan, Ping-Han Tsai, Ching-Hui Chen, Pi-Yueh Chang

**Affiliations:** From the Department of Internal Medicine, Division of Rheumatology, Allergy, and Immunology (K-HY, T-MC, P-HT); Department of Laboratory Medicine, Chang Gung Memorial Hospital, and Department of Medical Biotechnology and Laboratory Science (C-HC, P-YC); and Chang Gung University, Tao-Yuan, Taiwan (K-HY, P-YC).

## Abstract

The aim of this study is to study the clinical features and diagnostic performance of IgG4 in Chinese populations with IgG4-related diseases (IgG4-RDs).

The medical records of 2901 adult subjects who underwent serum IgG4 level tests conducted between December 2007 and May 2014 were reviewed.

Serum concentrations of IgG4 were measured in 2901 cases, including 161 (5.6%) patients with IgG4-RD and 2740 (94.4%) patients without IgG4-RD (non-IgG4-RD group). The mean age of the IgG4-RD patients was 58.4 ± 16.1 years (range: 21–87), and 48 (29.8%) were women. The mean serum IgG4 level was significantly much higher in IgG4-RD patients than in non-IgG4-RD (1062.6 vs 104.3 mg/dL, *P* < 0.001) participants. For IgG4 >135 mg/dL, the sensitivity, specificity, positive predictive value (PPV), negative predictive value (NPV), likelihood ratio (LR)+, and LR− were 86%, 77%, 18%, 99%, 3.70, and 0.19, respectively. When the upper limit of normal was doubled for an IgG4 >270 mg/dL, the corresponding data were 75%, 94%, 43%, 98%, 12.79, and 0.26, respectively. For IgG4 >405 mg/dL (tripling the upper limit of normal), the corresponding data were 62%, 98%, 68%, 98%, 37.00, and 0.39, respectively. When calculated according to the manufacturer's package insert cutoff (>201 mg/dL) for the diagnosis of IgG4-RD, the corresponding sensitivity, specificity, PPV, NPV, LR+, and LR− were 80%, 89%, 29%, 99%, 7.00, and 0.23, respectively. For IgG4 >402 mg/dL (>2× the upper limit of the normal range), the corresponding data were 62%, 98%, 68%, 98%, 36.21, and 0.39, respectively. For IgG4 >603 mg/dL (>3× the upper limit of the normal range), the corresponding data were 50%, 99%, 84%, 97%, 90.77 and 0.51, respectively. The optimal cutoff value of serum IgG4 (measured by nephelometry using a Siemens BN ProSpec instrument and Siemens reagent) for the diagnosis of IgG4-RD was 248 mg/dL, the sensitivity and specificity were 77.6% and 92.8%, respectively.

The present study demonstrated that 2 or 3 times the upper limit of the manufacturer's reference range of the IgG4 level was a useful marker for the diagnosis of various types of IgG4-RD and the optimal cutoff level was 248 mg/dL.

## INTRODUCTION

Immunoglobulin G4-related disease (IgG4-RD) is an inflammatory disorder with diverse clinical features including autoimmune pancreatitis (AIP), sclerosing cholangitis (SC), sclerosing sialadenitis, Mikulicz disease, retroperitoneal fibrosis, and many other disorders.^1–7^ Patients with IgG4-RD are characterized by tumefactive lesions in various organ systems or tissues, frequent elevations of the serum IgG4, and a rapid and good clinical response to steroid treatment.^1,2^ A diagnosis of IgG4-RD relies on the characteristics of biopsy specimens, including a lymphoplasmacytic infiltrate with abundant IgG4-positive plasma cells, storiform fibrosis, obilterative phlebitis, and eosinophilia.^1–4,6–8^

IgG4-RD has been identified as an emerging disease entity, and the diagnosis of this disease has increased rapidly in recent years. A high serum IgG4 level and increased numbers of IgG4-positive plasma cells in tissues have been regarded as important clues to the diagnosis of IgG4-RD.^1–3^ An elevated serum IgG4 concentration has been described as the hallmark of this condition with reported moderate-to-good sensitivity and specificity in the diagnosis of AIP and SC.^1–3^ However, wide variability exists in the reported frequency of elevated serum IgG4 levels in IgG4-RD,^3,5–31^ and the reliability of the serum IgG4 concentration as a marker for diagnosis has been questioned.^13,14,31^ Moreover, studies of the clinical features have often been small, focused on single-organ systems (particularly the pancreas)^3,8–10,12,17,20,21.24–28,30^ in Japanese^3,15–17,29,30^ or white participants^7–9,13,14,19,20^; the findings are not yet clear for a Chinese population.^11,12,26,27,30^ Furthermore, few studies have concentrated upon the diagnostic performance and cutoff of serum IgG4 levels for various IgG4-RD,^9,13,16,19,21,29^ and most such studies have been limited to patients with AIP or SC. The purpose of this investigation was to evaluate the diagnostic performance of IgG4 test in a real-world setting by reviewing the final diagnoses of patients who were tested in a tertiary medical center to evaluate their IgG4 level. We aimed to assess the best cutoff of serum IgG4 levels for the diagnosis of IgG4-RD in patients with a wide variety of clinical features and organ/tissue involvement.

## PATIENTS AND METHODS

All patients were treated at Chang Gung Memorial Hospital, a tertiary teaching medical center in Taiwan that has 3707 beds and admits over 150,000 patients each year. We identified patients with serum IgG4 levels measured between December 2007 and May 2014 and followed these patients up for at least 3 months. This study included 2901 adult subjects aged 18 years or older who underwent IgG4 testing at this institution. This study was approved by the Institutional Review Board of Chang Gung Memorial Hospital (CGMH, IRB-102–3061B). Written informed consent was not necessary due to the retrospective nature of this study. All blood specimens were sent to the clinical laboratory at CGMH, which is certified by the College of American Pathologists. Serum levels of IgG4 were determined by immunonephelometry using a Siemens BN ProSpec instrument and Siemens reagent (Siemens Healthcare Diagnostics, BN Prospec Nephelometer, Malburg, Germany). The upper limit of normal for the serum IgG4 level was 135 mg/dL, which was in accordance with most previous studies^3,15–17,19,22,28,29^ or was 201 mg/dL in accordance with the manufacturer's package insert (reference range: 3–201 mg/dL).^12^

The medical records of 2901 subjects were reviewed from the time of diagnosis until death, loss to follow-up, or the end of December 2014. The diagnosis of IgG4-RD was made according to clinical diagnosis and international guideline.^15,32^ Collected data included sex, age at onset, clinical features at presentation, laboratory test results from the first encounter, and mortality and morbidity. The data from the 161 IgG4-RD patients were used to compare the profiles of 2740 patients without IgG4-RD (non-IgG4-RD group).

Continuous variables are expressed as means ± SD. Categorical variables are shown as percentages and were analyzed with a *χ*^2^ test or Fisher exact test, and 2-tailed Student *t* test was used for group comparisons of numerical data. We evaluated the sensitivity, specificity, positive predictive value (PPV), negative predictive value (NPV), and likelihood ratio (LR) of elevated serum IgG4 concentrations for the diagnosis of various IgG4-RDs. The best cutoff values for IgG4 were determined by receiver-operating characteristic (ROC) and area under curve (AUC) analyses. A *P* value <0.05 was considered statistically significant. All statistical analyses were performed using SPSS software version 17.0. (SPSS Inc, Chicago, IL).

## RESULTS

Serum IgG4 levels were measured in the 2901 adult patients. Table 1 shows the characteristics of IgG4-RD patients stratified by organ/tissue involvement and other organ involvement (OOI). There were 161 patients (5.6%) diagnosed with IgG4-RD, and 2740 patients (94.4%) were diagnosed with other diseases (non-IgG4-RD group). The IgG4-RD group included 75 with type I AIP, 43 with IgG4-related ophthalmic disease, 12 with IgG4-related sialadenitis, 7 with IgG4-related lymphadenopathy, 6 with sclerosing angiomatoid nodular transformation, 4 with Mikulicz disease, 3 with IgG4-related retroperitoneal fibrosis, 3 with IgG4-related lung disease, 3 with IgG4-related SC, 3 with IgG4-related skin disease (including 1 case with Rosai-Dorfman disease), 1 with IgG4-related small intestinal disease, and 1 with IgG4-related periaortitis that presented with a huge pericardial tumor (Table 1). In 26 (16.1%) of the 161 patients, multiple organs were affected by IgG4-RD.

**TABLE 1:**
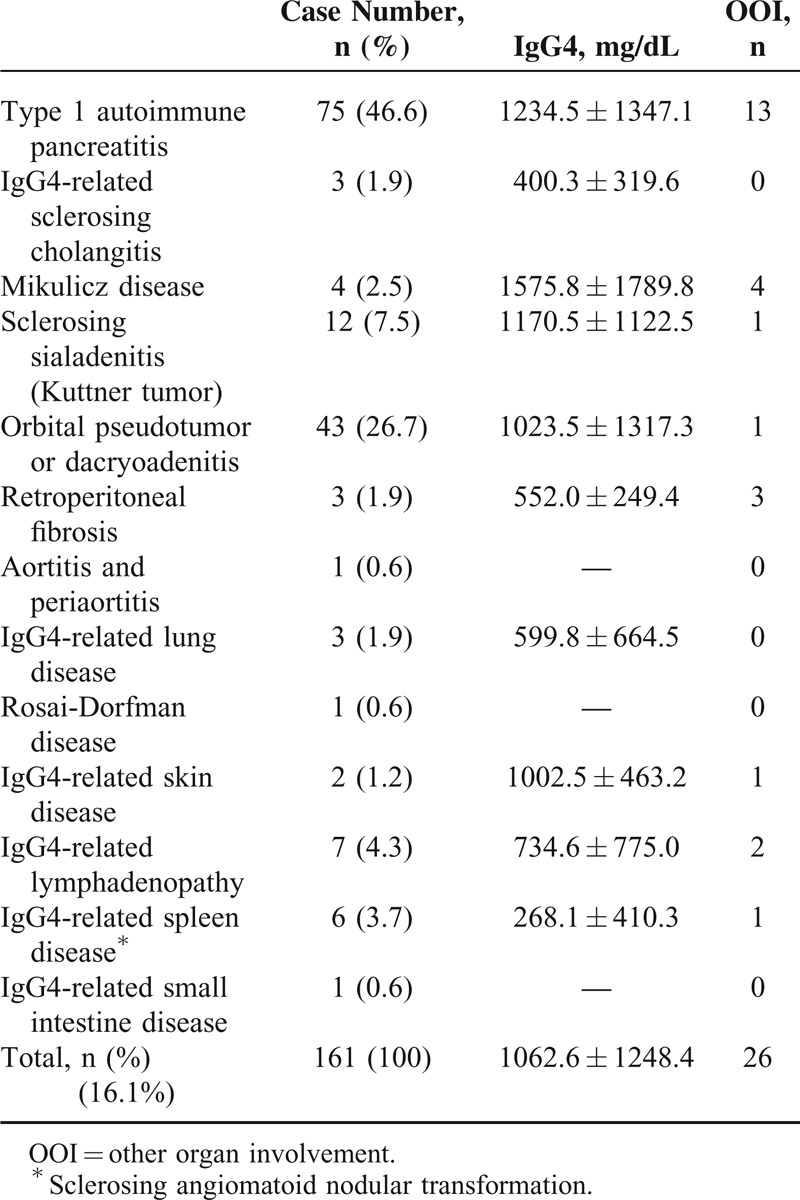
Clinical Manifestations of IgG4-Related Disease in 161 Chinese Patients Stratified By the Organ or Tissue Involved

Table 2 shows the percentage of elevated IgG4 levels in non-IgG4-RD groups. The non-IgG4-RD group included subjects diagnosed with pancreatic cancer (n = 199), pancreatitis (n = 459), cholangiocarcinoma (n = 63), cholangitis (n = 134), gastrointestinal malignancy (n = 51), lymphoma (n = 32), other malignancies (n = 286), allergic disease (n = 31), systemic lupus erythematosus (n = 109), rheumatoid arthritis (n = 433), Sjögren syndrome (n = 54), sarcoidosis (n = 2), systemic sclerosis (n = 5), vasculitis (n = 1), Castleman disease (n = 2), ankylosing spondylitis/psoriatic arthritis (n = 208), and other medical diseases (n = 671) (Table 2). Levels of serum IgG4 were elevated (>135 mg/dL) in IgG4-RD (138/161, 85.7%), hepatobiliary disorders (155/855, 18.1%), systemic autoimmune rheumatic disease (236/810, 29.1%), malignancy outside of the hepatobiliary system (76/369, 20.6%), allergic disease (13/31, 41.9%), Castleman disease (1/2), and miscellaneous medical illnesses (154/671, 23.0%). Overall, elevated serum IgG4 levels (>135 mg/dL) were noted in 23.2% (635/2740) of non-IgG4-RD patient groups and in 18.1% (36/199) of pancreatic cancer patients. However, elevated serum IgG4 levels (>201 mg/dL) were noted in 11.4% (311/2740) of non-IgG4-RD patient groups (Table 2). Subjects with IgG4-RD differed from those without IgG4-RD (non-IgG4-RD group) in mean age (58.4 ± 16.1 vs 53.5 ± 16.0 years, *P* < 0.001), men (70.2% vs 40.3%, *P* < 0.05), mean serum IgG4 level (1062.6 ± 1248.4 vs 104.3 ± 132.9 mg/dL, *P* < 0.001), and the presence of an elevated IgG4 >135 mg/dL (85.7% vs 23.2%, *P* < 0.001) or IgG4 >201 mg/dL (79.5% vs 11.4%, *P* < 0.001) (Table 3).

**TABLE 2:**
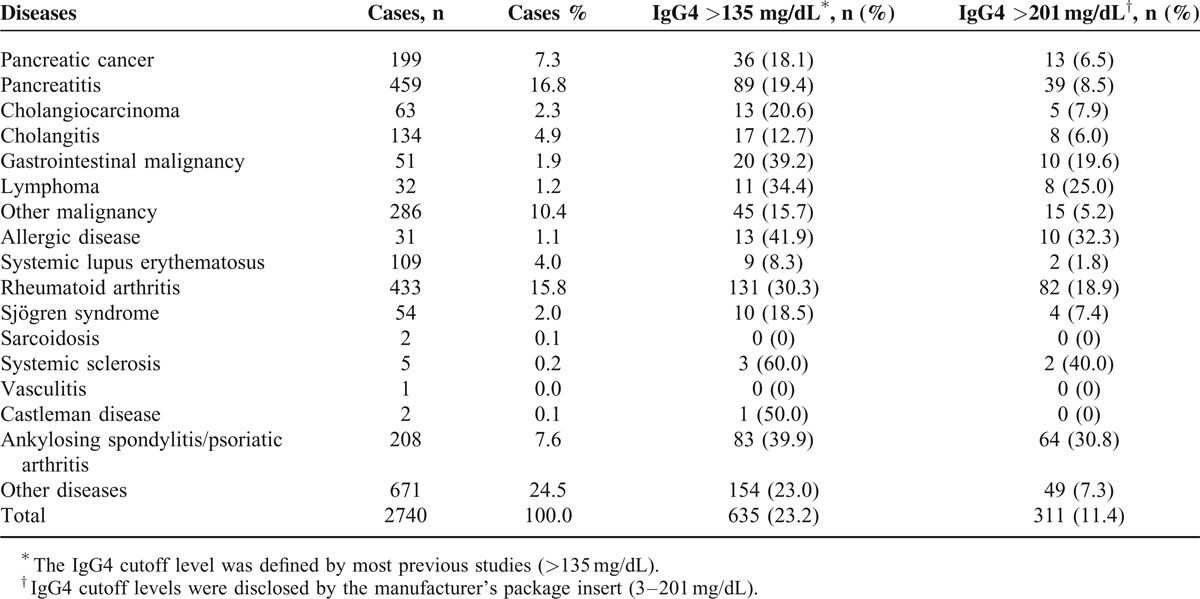
Characteristics of the 2740 Non-IgG4-RD Subjects and Range of Diseases With Elevated Serum IgG4 Levels

**TABLE 3:**
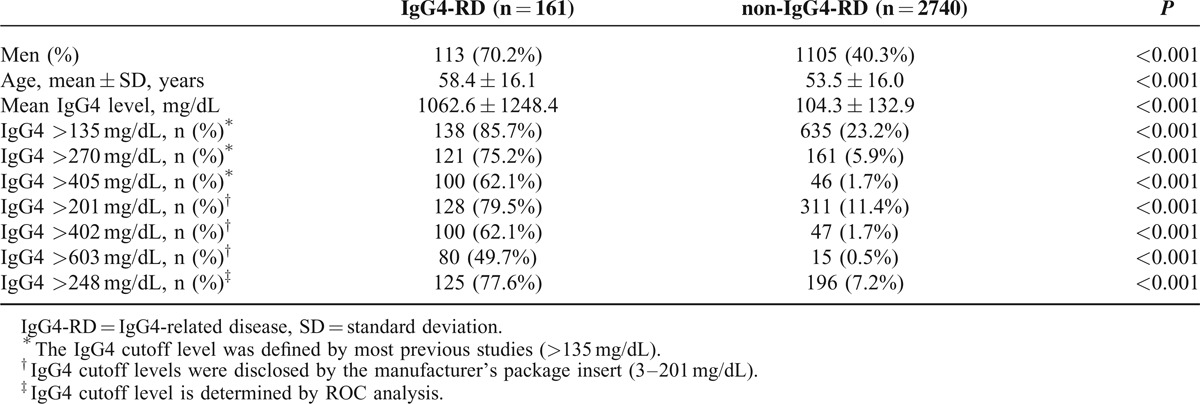
Demographics of Patients Both With and Without IgG4-RD Based Upon Different Serum IgG4 Cutoff Levels

**FIGURE 1:**
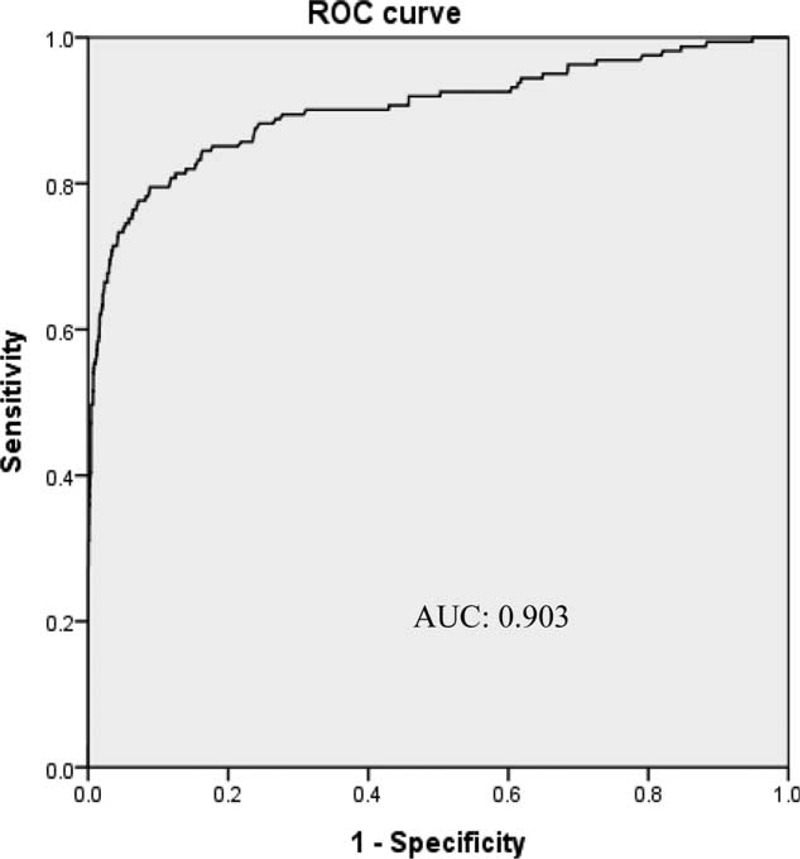
ROC curve for IgG4 levels in the diagnosis of IgG4-RD. AUC = area under curve, ROC = receiver-operating characteristic curve.

Because of the large number of non-IgG4-RD cases that were found to be associated with elevated IgG4 levels (23.2%, 635/2740), we evaluated the test performances of a cutoff value for the upper limit of normal for the serum IgG4 by ROC curves analysis. The optimal cutoff value of serum IgG4 (measured by nephelometry using a Siemens BN ProSpec instrument and Siemens reagent) for the diagnosis of IgG4-RD was 248 mg/dL, the sensitivity and specificity were 77.6% and 92.8%, respectively. The area under the ROC curve (AUC) for IgG4 level was 0.903 (95% confidence interval: 0.871–0.935, *P* < 0.001) (Fig. 1). In the diagnosis of IgG4-RD, the sensitivity, specificity, PPV, NPV, LR+, and LR− of the IgG4 level (>248 mg/dL) were 78%, 93%, 39%, 99%, 10.87, and 0.24, respectively. The sensitivity, specificity, PPV, NPV, LR+, and LR− of the traditional, most commonly used IgG4 level (>135 mg/dL) were 86%, 77%, 18%, 99%, 3.70, and 0.19, respectively (Table 4). The corresponding data for doubling the cutoff IgG4 level (>270 mg/dL) were 75%, 94%, 43%, 98%, 12.79, and 0.26, respectively. When the cutoff IgG4 level was increased to 3 times the normal reference value (>405 mg/dL), the corresponding data were 62%, 98%, 68%, 98%, 37.00, and 0.39, respectively. When using the manufacturer's reference cutoff (>201 mg/dL), the corresponding data were 80%, 89%, 29%, 99%, 7.00, and 0.23, respectively. When the cutoff IgG4 level was increased to twice the reference value of the manufacturer (>402 mg/dL, >2× the upper limit of the normal range), the corresponding data were 62%, 98%, 68%, 98%, 36.25, and 0.39, respectively. When the cutoff IgG4 level was increased to triple the reference value provided by the manufacturer (>603 mg/dL, >3× the upper limit of the normal range), the corresponding data were 50%, 99%, 84%, 97%, 90.77, and 0.51, respectively (Table 4).

**TABLE 4:**
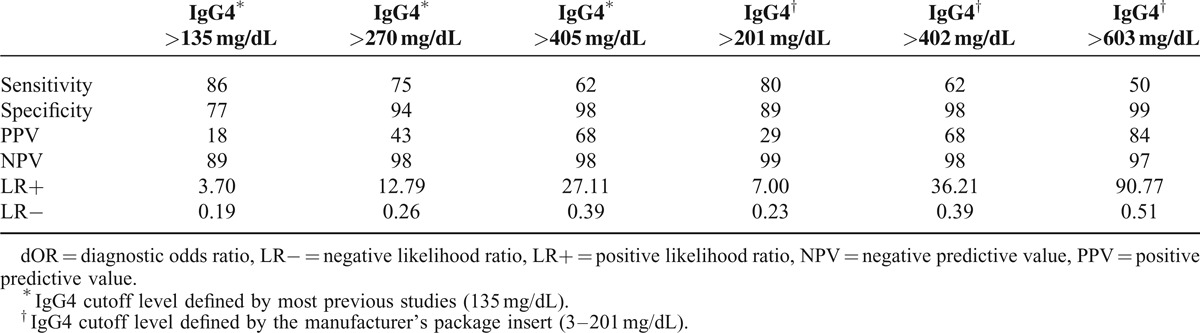
Performance of Serum IgG4 Levels in Patients With and Without IgG4-Related Disease, Stratified by Different IgG4 Cutoff Levels

## DISCUSSION

IgG4-related disease is a systemic disorder that may involve multiple organs and tissues, including the pancreas, bile duct, salivary gland, eye, lymph node, skin, retroperitoneum, aorta, pituitary gland, lung, liver, kidney, thyroid, breast, prostate, spleen, and other organs/tissues.^1–7^ The diagnosis of IgG4-RD depends primarily on clinical manifestations, imaging, and serological tests for the IgG4 levels. Diagnostic delays in the setting of IgG4-RD can lead to cirrhosis, pancreatic failure, aneurysms of the thoracic or abdominal aorta, advanced renal dysfunction, and many other complications.^1–4^ In the present study, we confirmed that nearly half of the diseases associated with IgG4-RD were AIP. In addition to AIP, more than half (53%) of the IgG4-related diseases were non-AIP IgG4-RD. Moreover, in the present study, 26 of the 161 (16.1%) IgG4-RD patients had OOI outside of the hepatobiliary system, which is also similar to previous studies.^5,8,10,12,13,17,20,22,33–35^ Ghazale et al^9^ studied 510 patients with various pancreatic diseases, of whom only 45 had AIP (8.8%), which is similar to the present study's findings (8.3%, 75/906 hepatobiliary disorders; or 5.6%, 161/2901 total cases).

Moreover, the present study revealed that patients with elevated serum IgG4 levels encountered in clinical practice manifest a wide range of diseases (Table 2). Although the serum IgG4 level has been described to be the most sensitive and specific laboratory test for the diagnosis of IgG4-RD, it is also recognized that an elevated serum IgG4 level can be encountered in other diseases, including Churg-Strauss syndrome, multicentric Castleman disease, sarcoidosis, pemphigus, allergic disorders, chronic hepatitis, liver cirrhosis, and systemic autoimmune rheumatic diseases, such as rheumatoid arthritis, systemic sclerosis, and Sjögren syndrome.^1–4,29,36–42^ In the present study, we confirmed that autoimmune rheumatic diseases are likely under-recognized diseases associated with IgG4 elevation.^29^ However, in the setting of autoimmune rheumatic diseases, elevated serum IgG4 levels are not specific for IgG4-RD and are likely to be false-positives in the absence of clinical evidence of IgG4-RD. Of note, a long-term follow-up study of patients with autoimmune rheumatic disease demonstrated no increased risk of developing IgG4-RD compared with the general population.^29^ In a population with low pretest probability (ie, prevalence) of IgG4-RD, the serum IgG4 concentration has a poor PPV for the diagnosis of IgG4-RD and, therefore, the clinical significance of elevated serum IgG4 concentration alone must be interpreted with caution.

IgG4-RD is a chronic fibroinflammatory condition frequently associated with elevations in serum IgG4 levels and tissue infiltration with abundant IgG4 + plasma cells. Accumulating evidence has confirmed that the IgG4 level is useful in the diagnosis of IgG4-RD. In previous studies and meta-analyses, the reported sensitivity of IgG4 for the diagnosis of IgG4-RD ranged from 52% to 97%,^3,8–11,12,14,16,19,21,22,28,29^ whereas the specificity ranged from 60% to 97%.^3,9,11,16,19,21,22,28,29^ The use of IgG4 for the diagnosis of AIP was first suggested in 2001^3^; Hamano et al found that elevated serum IgG4 levels were 95% sensitive and 97% specific in differentiating AIP from pancreatic cancer.^3,9^ Subsequent large cohorts of patients with a wide variety of pancreatic diseases showed that elevated serum IgG4 levels are characteristic but not diagnostic of AIP.^9,16,17,19,21^ Kamisawa et al^28^ reported that elevated serum IgG4 levels in 58% to 100% of AIP cases and the mean level ranged from 336.8 to 729.3 mg/dL. In the present study, the majority of IgG4-RD patients had elevated serum concentrations of IgG4 (80% >201 mg/dL and 86% >135 mg/dL). Among the 161 patients with diagnoses of IgG4-RD, only 23 (14.3%) had normal serum IgG4 concentrations (≤135 mg/dL). Moreover, the present study revealed that patients with elevated serum IgG4 levels encountered in clinical practice manifest a wide range of diseases, and only 18% (138/773) of them have IgG4-RD, which is similar to the findings of previous studies in whites^5,9,13^ and Japanese.^16^ Studies have highlighted the spectrum of disorders associated with elevated serum IgG4 levels and concluded that a majority of patients with serum IgG4 elevations do not have IgG4-RD. In this study, >82.1% (635/773) of subjects with elevated IgG4 levels (>135 mg/dL) showed no evidence of an IgG4-RD. The corresponding value for a cutoff point of IgG4 >201 mg/dL based upon the manufacturer's package insert was 70.8% (311/439). Despite these shortcomings, the large proportion of patients with elevated IgG4 levels without evidence of the features of an IgG4-RD is an important observation.^5,9,13,16^

In previous studies, the upper limit of normal (ULN) for serum IgG4 is considered to be 140 mg/dL according to the Mayo Clinic criteria^5–9,14,43^ and 135 mg/dL based upon the Comprehensive Diagnostic Criteria,^29^ revised clinical diagnostic criteria for type 1 AIP (JPS-2011),^44,45^ and clinical diagnostic criteria for IgG4-SC (IgG4-SC-2012),^46^ whereas some use a cutoff of 130 mg/dL^14,20^ or even 119 mg/dL.^16^ The HISORt criteria from the Mayo Clinic^8,43^ and the Japanese consensus criteria^43^ were mainly produced to facilitate the diagnosis of Type I AIP. Chari et al^8^ introduced the HISORt criteria based on a prospective study of 29 consecutive patients at the Mayo Clinic who met the histological criteria for AIP. The Mayo Clinic reported a sensitivity, specificity, and positive predictive value of 76%, 93%, and 36%, respectively, using a cutoff value for IgG4 of 140 mg/dL.^9^ A serum IgG4 level >2 times the upper limit of normal greatly increased the specificity (99%) for AIP,^9^ which is a similar finding as that of the present study. It is noteworthy that the revised HISTOR criteria^43^ and the International Consensus Diagnostic Criteria for AIP^47^ consider an IgG4 level more than twice the upper limit of normal to be highly suggestive. Moreover, an elevation of the serum IgG4 over 2 or 3 times the upper limit of normal is generally considered to be specific and highly suggestive (or diagnostic) for AIP and IgG4-RD.^48^

The diagnostic performance of IgG4 in a Chinese population in the present study is similar to a previous meta-analysis, which evaluated the usefulness of serum IgG4 in diagnosing AIP and showed variations in sensitivity and specificity ranging from 67% to 94% and 89% to 100%, respectively.^11^ In previous studies,^3,5–29^ important differences were seen in the characteristics of patients evaluated as well as the cutoff value used to define a positive test. Previous studies on serum IgG4 measured in targeted populations with a high probability of having one or another manifestation of IgG4-RD have reported a high sensitivity and specificity.^3,9,11,16,17,19,22,29^ High serum IgG4 levels are reported to have a moderate-to-good sensitivity and specificity for the diagnosis of IgG4-RD but because of the low prevalence of the condition, the PPV of the serum IgG4 test is reported to be between 10% to 36% depending on the study populations.^9,13,14,16,19^ Consistent with previous studies, the findings of this study confirm that the PPV for the diagnosis of IgG4-RD is low at 17.9% with a cutoff of 135 mg/dL, which is similar to a previous study of 15% with a cutoff of 130 mg/dL^14^; however, the prevalence of IgG-RD in that study was very low (0.72%, 9/1258)^12^ in contrast to the prevalence of IgG4-RD in the present study (5.6%, 161/2901). Similarly, another 2 studies from the Mayo Clinic showed the PPV for IgG4-RD was 18.4% (29/158)^5^ and 10.0% (39/390),^13^ but the prevalence (ie, pretest probability) of IgG4-RD was less than 1% (0.88%, 29/3300;^5^ and 0.65%, 39/6014,^13^ respectively). In contrast, the prevalence of AIP in the study by Ghazale et al^9^ was 8.8% (45/510), the PPV was 36% (>140 mg/dL), and the PPV could be improved to 75% if a serum IgG4 level of 280 mg/dL (2 times the upper limit of the reference range) was used, which is similar to the present study (43% for 2 times the upper limit of the most commonly used cutoff [>270 mg/dL], or 68% for 2 times the upper limit of the manufacturer's reference range [>402 mg/dL]). Another study from Japan has also come to similar conclusions.^16^ These studies^9,16^ have a higher proportion of patients with IgG4-RD (5.6%–12.1%) and subsequently can offer a much higher positive predictive value. However, in another 2 previous studies,^5,13,14^ the pretest probability (prevalence of targeted disease) was low (0.7%–0.9%); thus, the PPV is much lower. These differences might explain the wide range of sensitivity, specificity, and PPV results reported.^5,9,13,14,16,19^ The present study demonstrated that serum IgG4 concentration has valuable diagnostic utility when the pretest probability of the disease is more than several percent; therefore, indiscriminate testing of serum IgG4 levels should be discouraged as it does not add diagnostic value in the absence of consistent clinical findings. Moreover, a serum IgG4 >135 mg/dL has been widely accepted as the cutoff value for the diagnosis of IgG4-RD. However, there is no agreed-upon international standard for IgG4 quantification or the IgG4 cutoff level.^31,47,48^ The best IgG4 cutoff in the present study (measured by nephelometry using a Siemens BN ProSpec instrument and Siemens reagent) was 248 mg/dL for IgG4-RD. It was noted that the method and reagent used for IgG4 testing requires special concern as there was a 2-fold difference in the IgG4 levels obtained by different methods and reagents used for IgG4 testing.^31,48^ The differences in calibration reflected in the stated adult reference ranges for the 2 companies should be noted when interpreting the IgG4 data: for the Binding Site, 3.9–86.4 mg/dL, and for Siemens, 3.0–201 mg/dL.^31^

There are limitations of the present study that warrant discussion. First, it is still possible that some cases of IgG4-RD may have been missed and misclassified, especially in patients who did not have a classic IgG4-RD presentation. However, this problem is common to all studies that did not use tissue pathology-proven cases as the criterion standard of IgG4-RD. Second, problems might occur due to the fact that for the definitive diagnosis, histology is required, which often is difficult to obtain (eg, from the retroperitoneum, the orbita, or the pancreas). Thus, the probability of misclassification is possible. However, the present study investigated a large number of unselected patients that had been enriched for patients with multiorgan as opposed to single-organ disease and included AIP and non-AIP IgG4-RD. One strength of this study was that we investigated the clinical utility of an IgG4 test performed not only outside gastroenterology setting but that covered all ranges of IgG4-RD. Moreover, this study investigated the clinical utility of different cutoff IgG4 levels in a real-world setting of serum IgG4 levels in 2901 Chinese patients.

In conclusion, the serum IgG4 concentration has a moderate-to-good positive predictive value in the diagnosis of IgG4-related sclerosing disease when it is more than 2 or 3 times the ULN of the manufacturer's reference. Furthermore, a number of conditions may also result in high IgG4 concentrations and should be kept in mind when interpreting the IgG4 level. Failure to understand the test characteristics of serum assays for IgG4 and to employ them effectively in clinical practice can lead to either over diagnosis or delays in diagnosis. Characteristic histology and response to steroids can further establish the diagnosis of IgG4-RD in patients either with or without elevation of serum IgG4 levels. When reporting results, it would be helpful for laboratories to include their in-house data on the background rate of IgG4 positivity because many practicing clinicians are not aware of the high background rate of IgG4 positivity in the general population and the normal value of IgG4 levels varies by different methods and reagents used for IgG4 testing.
